# Individualized treatment with infliximab therapy in children with Crohn’s disease support shorter time intervals between infusions

**DOI:** 10.3109/21556660.2012.655815

**Published:** 2012-01-09

**Authors:** M. Sommar, S. Eksborg, H. Hildebrand, L. Grahnquist

**Affiliations:** 1 Karolinska Pharmacy, Karolinska University Hospital, StockholmSweden; 2 Karolinska Pharmacy, Karolinska University Hospital, Stockholm and Department of Women’s and Children’s Health, Karolinska Institutet, StockholmSweden; 3 Department of Women’s and Children’s Health, Karolinska Institutet, Stockholm and Astrid Lindgren Children’s Hospital, Karolinska University Hospital, StockholmSweden

**Keywords:** Children, Clinical outcome, Crohn’s disease, Infliximab, Maintenance therapy

## Abstract

**Objectives:**

To study the effect of an individualized treatment approach with regard to dosage intervals between infliximab infusions on the clinical outcome of pediatric Crohn’s disease (CD).

**Patients and methods:**

A retrospective analysis of medical records of all pediatric patients with CD who had been treated with infliximab between 1999 and 2007 in two Swedish counties, where an individualized treatment approach had been applied.

**Results:**

Twenty-nine patients were included in the study. The number of infusions varied from 2 to 47 (median: 8). Nineteen patients received more than 5 infusions and 13 patients received more than 10 infusions. Most of the patients did not stay in remission when the dosage interval was 8 weeks or longer.

**Conclusions:**

An individualized treatment approach, based on the physician’s desire to treat, resulted in shorter dosage intervals than 8 weeks between infliximab infusions in a majority of pediatric patients with CD. The retrospective design of the study must be taken into account when interpreting the results.

## Introduction

The incidence of pediatric Crohn’s disease (CD) increased during the late 1990s in many European countries^1–5^. Medical treatment of CD has mainly consisted of corticosteroids, 5-acetylsalicylic acid compounds, immunosuppressants and antibiotics. There are benefits and drawbacks to all these groups of medication^6–9^. Anti-TNF agents are the latest contribution to the treatment of CD, where infliximab has proven to be efficient in the adult population^10^.

The efficacy of infliximab in children with Crohn’s disease (CD) has been documented in several studies^11–13^, but the experience in children is still limited. All published results are based on an induction regimen of infusions at weeks 0, 2 and 6, followed by maintenance therapy with longer intervals between infusions. Maintenance therapy has either been given as scheduled therapy or as on-demand therapy. A higher remission rate in patients treated with maintenance infliximab therapy every 8 weeks compared with patients treated every 12 weeks was demonstrated in the largest study so far, the REACH study^11^.

It has not yet been demonstrated that the pediatric patients have been free from symptoms during the entire time interval between the infliximab infusions. A trend towards shorter time intervals between infusions of infliximab in some pediatric patients with CD was reported in a study from 2003^14^. An optimum maintenance therapy strategy to increase the efficacy of infliximab is still needed, as it appears that a number of patients need modified infusion protocols^14,15^. In the present retrospective study we recorded the outcome of an individualized dosage regimen during maintenance infliximab therapy in children with CD.

## Patients and methods

A retrospective review was carried out of the medical records of all pediatric CD patients treated with infliximab in Stockholm and Södermanland County in Sweden between March 1999 and September 2007. Data were collected from hospitals in five different locations (Solna, Huddinge, Södermalm, Eskilstuna and Nyköping).

All patients treated with infliximab and diagnosed with CD were included in the study. Only patients receiving more than three infusions were eligible for an individualized schedule. All patients were scheduled to an induction regimen of three infusions prior to individualized therapy according to the infusion protocol.

Following the third infusion and during maintenance therapy, a strategy was applied to identify an optimum time between infusions. This was based on the time in remission since the last infusion, with the aim of stretching the time interval further if possible. Consequently, the strategy was to give the next infusion before symptoms recurred. The decision when to treat patients was left to the physician with the instruction to give the next infusion before time of flare. Patients were instructed to contact the treating physician when minimum signs and symptoms of relapsing disease was evident, even when between two regular visits at the clinic. A lower limit of 4 weeks but no upper limit regarding time between infusions was set.

The efficacy of infliximab from the time of the patient’s last infusion to the next infusion was quantified using the terms ‘remission’ and ‘clinical response’. ‘Remission’ referred to total loss of symptoms, whereas ‘clinical response’ referred to an improved clinical status after the last infliximab infusion but not to a total loss of symptoms. A patient was considered to be in ‘remission’ or showing a ‘clinical response’ if these criteria were fulfilled during more or equal to 50% of the time between infusions. ‘Loss of response’ was used to define a situation when the clinical symptoms for which infliximab was used returned before the subsequent infusion was due, and when shortening the time interval was insufficient to control the symptoms.

Results are presented as median and range.

## Results

### Patient data

A total of 29 patients, 20 boys and nine girls were treated during the study period. The median age at diagnosis was 12.4 years (range: 3.2–16.9 years). At the start of infliximab therapy the median age was 15.3 years (range: 8.1–18.7 years). The time between diagnosis and the start of infliximab therapy ranged from 2 (in a patient with concomitant arthritis) to 69 months (median: 29 months). The time of treatment ranged from 1 to 76 months (median: 11 months).

All patients were on treatment with at least one other drug (immunosuppressant, antibiotic, steroid or 5-acetylsalicylic acid-related compound) at the start of infliximab therapy.

Four patients, two of whom were followed-up, had been referred to adult centers for further treatment after starting infliximab therapy. Another four patients were transferred to other hospitals, one of whom was followed-up. One patient moved abroad and was lost to follow-up.

### Infusions of infliximab

A total of 333 infusions of infliximab were given (median number of infusions per patient: 8, range: 2–47). Ten patients received less than 6 infusions. Six patients received 6–10 infusions, seven patients received 11–15 infusions and six patients received more than 15 infusions.

**Figure 1.**
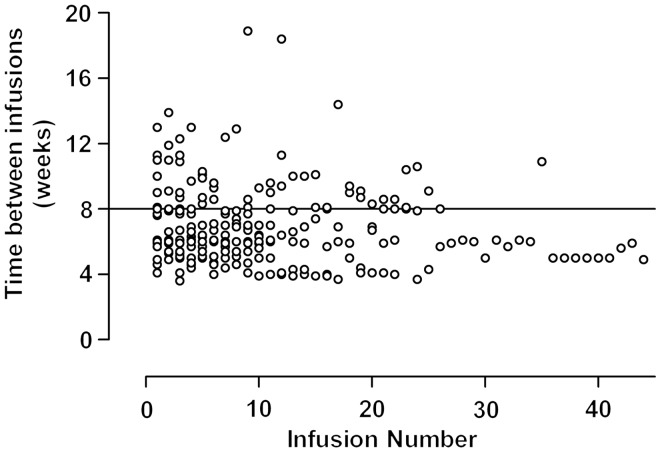
All infusions given to patients eligible for individualized therapy.

All but one patient (due to an anaphylactic shock) received an induction regimen of three infliximab infusions, with the second and third infusions given approximately 2 and 6 weeks, respectively, after the first infusion. Some patients received their induction infusions with other time intervals due to active infections or practical difficulties at the time of a planned infusion. The median time between the first and second infusion was 2.5 weeks (range: 1.9–13.1 weeks) and between the second and the third infusion 4.0 weeks (range: 2.9–21.0 weeks). The time between infusions in the 22 patients who received more than three infusions of infliximab and who were eligible for an individualized schedule are shown in Figure 1. There was a great variability in dosage intervals during the study period, with a trend towards shorter time intervals over time. Out of 183 infusions, 59 (32%; 95% CI: 26–39%) were given at an interval of 8 weeks or more, and 127 infusions (68%; 95% CI: 61–75%) were given at an interval of less than 8 weeks.

### Efficacy

**Figure 2.**
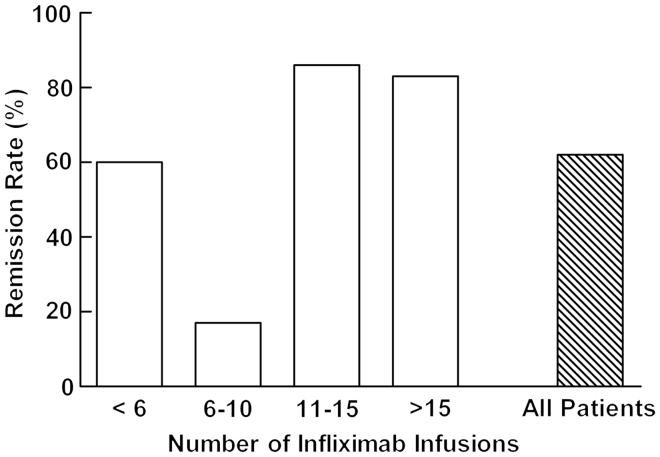
Remission rates of all groups of patients.

In all, 62% of the patients reached ‘remission’ with infliximab treatment (Figure 2). In the subgroup of patients who received more than ten infusions, 85% reached ‘remission’ on infliximab treatment during the study period. A lower response rate to treatment was seen in the group of patients receiving 6–10 infusions where five of six patients stopped treatment. In this group one patient suffered from an anaphylactic shock and one developed genital herpes zoster, which led to therapy termination. Three patients stopped treatment due to ‘loss of response’. In all three patients, the dosage interval was shortened before withdrawal of therapy.

## Discussion

This retrospective study of children with CD indicates that, when the decision to maintain successful long-term outcome of infliximab therapy was left to the physician, a majority of patients required shorter time intervals than 8 weeks as suggested in the ordinary treatment protocol.

Recent studies, prospective as well as retrospective, show various strategies for infliximab use in the treatment of CD. Maintenance therapy has either been given as scheduled^11^, on demand^14^, or through a combination of both strategies^13,15^ and have all demonstrated the efficacy of infliximab. A study on the single-dose regimen showed temporary good clinical efficacy, apart from in a few patients who had sustained good efficacy when treated early after diagnosis^16^. Good clinical efficacy was observed after the induction regimen only, especially in patients with anal fistulae, but 90% of the patients relapsed within 1 year^17^. However, the importance of a proper dosing interval to obtain good efficacy of infliximab has not fully been outlined.

The present study was performed in order to investigate the outcome of individualized treatment approach with regard to the long-term outcome of infliximab in children with CD. The induction regimen was largely performed according to the manufacturer’s recommendation at weeks 0, 2 and 6, respectively. The efficacy of infliximab was comparable with the REACH study with the same level of ‘remission’ after the induction treatment^11^. During maintenance therapy, our strategy was to give the next infusion before symptoms occurred, leaving the decision when to treat patients to the physician. As a result of this, a significantly higher proportion of infusions was given with intervals less than 8 weeks compared to 8 weeks or more during the study period. Our results indicate that the long-term efficacy outcome of infliximab in a subset of children was improved or maintained during maintenance therapy when shorter infusion intervals than 8 weeks were used. This was not applicable to all patients, as a number of patients stopped treatment even though individualized treatment was tried. Improved clinical efficacy of infliximab with shorter intervals between infusions than 8 weeks in a subset of patients has also been shown by Stephens *et al*. in 2003 in young patients (age 5–23 years) with CD, albeit without any statistical support for their findings^14^. In our present study, 85% of the patients who received more than ten infusions of infliximab reached ‘remission’ on treatment and constitute a selected group of patients for whom the risk of dropout has been very low. Results similar to ours have also been demonstrated in an adult population where the majority of patients with a previous loss of response to infliximab treatment regained their response after dose intensification or a shortening of the infusion intervals^18,19^.

The retrospective nature of this study needs to be acknowledged as a limitation. PCDAI was not unanimously used during patient evaluation; instead, we used ‘remission’ and ‘clinical response’. This may have resulted in judgments being varied on when to treat patients. It is also possible that some patients waited too long before getting in touch with the physician, resulting in a clinical relapse before being treated. In a randomized study comparing scheduled to on-demand therapy, patients receiving the scheduled regimen experienced better disease control^20^. In this study a Harvey–Bradshaw Index^21^ of ≥5 was a criterion for receiving further treatment for the patients receiving on-demand therapy, which may explain the discrepancy. Further, we did not analyze patients with fistulas separately due to the small sample size. For the same reason patients receiving infliximab monotherapy or different combinations of other concomitant treatments were not analyzed separately. Dose intensification was attempted for a minority of patients, but this was not considered to affect the results.

**Table 1.** Indications for therapy.

Therapy-resistant CD only	13
Fistulae only	6
Arthritis main indication for treatment*	1
Therapy-resistant CD + fistulas	6
Therapy-resistant CD + arthritis	1
Therapy-resistant CD + fistulas + arthritis	1
Growth retardation + steroid dependency	1
Total	29

*This patient was initially treated for arthritis, but later on also for CD.

In conclusion, our data are the first to indicate that long-term outcome regarding efficacy of infliximab is for a majority of patients dependent on shorter dosage intervals in children with CD. This study implies that an individualized treatment approach is possible and may lead to a better patient care in routine clinical practice. Further studies should address whether there are subgroups of patients that can become free from medication for a longer time period after having reached remission during this treatment approach.

## Transparency

### Declaration of financial/other relationships

None of the authors have any conflicts of interests. H.H. and L.G. are pediatricians employed by Karolinska University Hospital. S.E. is a pharmacologist employed by Karolinska University Hospital. The authors declare no conflict of interests.
